# Effects of isoflurane, sevoflurane, propofol and alfaxalone on brain metabolism in dogs assessed by proton magnetic resonance spectroscopy (^1^H MRS)

**DOI:** 10.1186/s12917-018-1396-1

**Published:** 2018-03-05

**Authors:** Franz Josef Söbbeler, Inés Carrera, Kirby Pasloske, Millagahamada Gedara Ranasinghe, Patrick Kircher, Sabine Beate Rita Kästner

**Affiliations:** 1 Small Animal Clinic (Söbbeler, Kästner), University of Veterinary Medicine Hanover Foundation, Bünteweg 9, 30559 Hannover, Germany; 2 Clinic of Diagnostic Imaging (Carrera, Kircher), Vetsuisse Faculty, University of Zurich, Winterthurestrasse 258c, 8057 Zurich, Switzerland; 3 Jurox Pty Ltd. (Pasloske, Ranasinghe), 85 Gardiner St, Rutherford, NSW 2320 Australia

**Keywords:** Anesthesia, Lactate, Glucose, MRI, Cerebral, PRESS, Plasma, LcModel, Canine

## Abstract

**Background:**

The purpose of this study was to determine the effects of isoflurane, sevoflurane, propofol and alfaxalone on the canine brain metabolite bioprofile, measured with single voxel short echo time proton magnetic resonance spectroscopy at 3 Tesla. Ten adult healthy Beagle dogs were assigned to receive isoflurane, sevoflurane, propofol and alfaxalone at 3 different dose rates each in a randomized cross-over study design. Doses for isoflurane, sevoflurane, propofol and alfaxalone were F_E_’Iso 1.7 vol%, 2.1 vol%, 2.8 vol%, F_E_’Sevo 2.8 vol%, 3.5 vol% and 4.7 vol%, 30, 45 and 60 mg kg^− 1^ h^− 1^ and 10, 15 and 20 mg kg^− 1^ h^− 1^ respectively. A single voxel Point Resolved Spectroscopy Sequence was performed on a 3 T MRI scanner in three brain regions (basal ganglia, parietal and occipital lobes). Spectral data were analyzed with LCModel. Concentration of total N-acetylaspartate (tNAA), choline, creatine, inositol and glutamine and glutamate complex (Glx) relative to water content was obtained. Plasma concentration of lactate, glucose, triglycerides, propofol and alfaxalone were determined. Statistics were performed using repeated measures ANOVA or Wilcoxon Sign Rank test with alpha = 5%.

**Results:**

Plasma glucose increased with isoflurane, sevoflurane and alfaxalone but decreased with propofol. Plasma lactate increased with all anesthetics (isoflurane > sevoflurane > propofol > alfaxalone). Cerebral lactate could not be detected. Only minor changes in cerebral metabolite concentrations of tNAA, choline, inositol, creatine and Glx occurred with anesthetic dose changes.

**Conclusion:**

The metabolomic profile detected with proton magnetic resonance spectroscopy at 3 Tesla of canine brain showed only minor differences between doses and anesthetics related to tNAA, choline, creatine, inositol and Glx.

## Background

Injectable as well as volatile anesthetics influence cerebral metabolism. The classic paradigm that neuronal activity ≈ metabolism ≈ blood flow has been used to study the effect of anesthetics on brain metabolism. Positron Emission Tomography studies with 2-deoxy-2(^18^F)fluoro-D-glucose to determine the cerebral metabolic rate of glucose (CMRglu) as an indirect measure of neuronal activity [1] found a consistent suppression of CMRglu under isoflurane, sevoflurane and propofol with varying impact on different brain regions in man [2–6]. Cerebral metabolic rate of oxygen is also reduced by sevoflurane, propofol, isoflurane and also Althesin® (a combination of 9 mg mL^− 1^ alfaxalone- and 3 mg mL^− 1^ alfadolone acetate) [7–9]. In microdialysis studies in rodents [10, 11] volatile anesthetics led to a rapid and dose-dependent increase in cerebral lactate levels without changes in cerebral glucose levels. Ketamine/xylazine, and chloral hydrate moderately increased cerebral lactate, but cerebral glucose levels rose markedly. Both propofol and pentobarbital did not affect cerebral lactate levels and only propofol led to a minor increase in cerebral glucose.

Brain metabolism can also be investigated by magnetic resonance spectroscopy (MRS), which is a non-invasive magnetic resonance imaging technique that allows the determination of the biochemical composition of the brain in vivo for accurate identification and quantification of metabolites in localized brain regions [12–14]. With proton magnetic resonance spectroscopy (^1^H MRS) at 3 Tesla several metabolites can be identified in the normal brain, such as N-acetylaspartate (NAA), choline, creatine, myo-inositol, and sum of glutamate and glutamine (Glx) [15–18]. At > 7 Tesla, the spectral differentiation between the glutamine and glutamate is complete, and other smaller metabolites like glucose and GABA are well depicted [19, 20].

Several ^1^H MRS studies have been performed to investigate metabolism within the brain under general anesthesia in rodents [21, 22], monkeys [23]*,* humans [24] and dogs [25]. The main findings of these studies [21–24] were the increase in cerebral lactate under the dose-dependent influence of volatile anesthetics independent of plasma lactate [21–23]. They also found increases in other cerebral metabolites, especially glutamate, alanine and creatine. These changes have been shown to be fully reversible within minutes after completion of the anesthesia [21]. On the other hand, the injectable anesthetics and premedication drugs ketamine/medetomidine, medetomidine, pentobarbital, fentanyl, diazepam and propofol did not influence cerebral lactate concentration [21, 22].

In veterinary medicine, clinical MRI can only be performed under general anesthesia. Recently, relative metabolite concentrations and their ratios in the brain of healthy beagle dogs under general anesthesia have been published [15–17], as well as a few clinical studies [26, 27] using different anesthetic protocols. Since the influence of anesthetics on the canine brain bioprofile at 3 Tesla is actually not known, the purpose of the study reported here was to determine the possible effects of the commonly used anesthetics isoflurane, sevoflurane, propofol and alfaxalone on canine brain bioprofile assessed with single voxel short echo time proton magnetic resonance spectroscopy at a field strength of 3.0 Tesla. Secondary aim of this study was to evaluate the possible effects of the anesthetics on plasma levels of lactate and glucose.

Our hypothesis was that volatile anesthetics isoflurane and sevoflurane, but not the injectable anesthetics propofol and alfaxalone, cause a dose-dependent increase in cerebral lactate. Furthermore, we hypothesized an increase in cerebral levels of creatine and glutamate under influence of volatile anesthetics compared to the injectable anesthetics propofol and alfaxalone. Regional differences in cerebral metabolites were anticipated to be independent of anesthetics.

## Methods

All animal procedures were performed according to the German animal protection law after review and approval by the ethical committee for animal experimentation of the Federal State Office for Consumer Protection and Food Safety of Lower Saxony, Germany (3392 42,502–04-13/1252).

### Animals

Ten purpose-bred (purpose bred at our own institution) adult Beagle dogs (8 neutered males and 2 spayed females) were included in the study. Age ranged from 32 to 91 month (mean ± SD, 50.7 ± 22.2 months) and the weight ranged from 11 to 21.8 kg (mean ± SD, 16.5 kg ± 3.2).

The dogs were housed in groups of 5 to 6 dogs per group with daily periods in enriched outside runs and regular walking by veterinary students. Ambient temperature is regulated to 18–21 °C. The dogs received commercial dog food (Hills VetEssentials Canine adult) according to body weight, twice daily except on study days.

Health status of the dogs was confirmed by a clinical and neurological examination and complete blood count and blood chemistry. Physical status was classified according to the American Society of Anesthesiologists (ASA) physical status classification system as ASA I.

### Study design

The study was performed as a randomized, experimental trial in a complete cross-over design with four treatments [isoflurane (I), sevoflurane (S), propofol (P), alfaxalone (A)] and a wash out period of at least 14 days between treatments.

### Instrumentation

All dogs were fasted overnight, water was available until the trial started. Blood was drawn from the jugular vein for analysis of blood gases, electrolytes, plasma lactate, plasma glucose and plasma triglycerides. A peripheral venous catheter was placed into the cephalic or lateral saphenous vein for induction and maintenance of anesthesia (treatment P and A) and cardiovascular support (treatment I, S, P, A). A central venous catheter was placed into the right or left jugular vein for blood sampling. All dogs were wrapped in self-made bubble wrap vests equipped with 41 °C warm gel heat pads for temperature management. Earplugs were used for noise protection.

### Anesthetic protocol

**Fig. 1:**
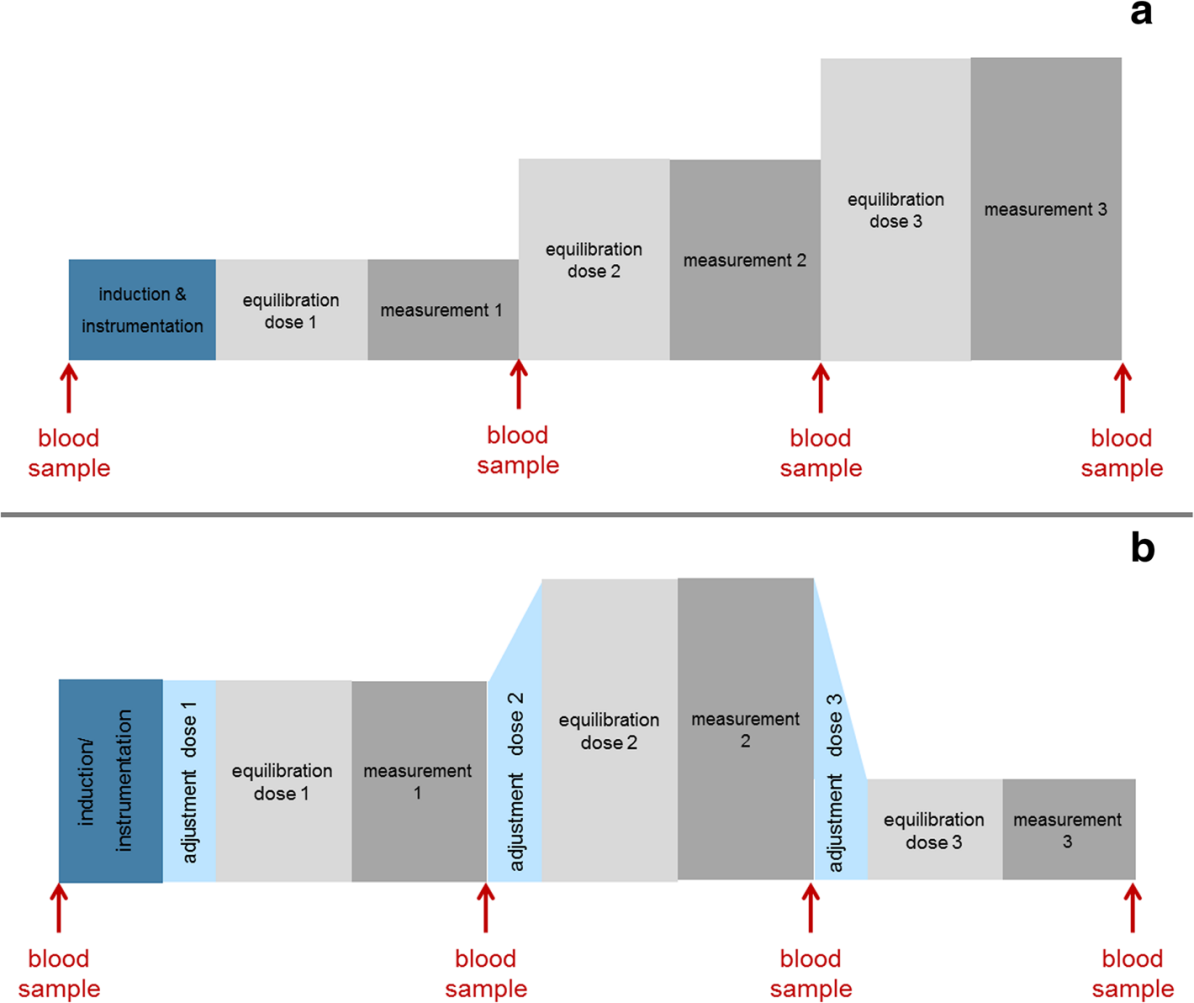
Schematic timeline for propofol and alfaxalone (**a**) and isoflurane and sevoflurane (**b**) The 30 min equilibration phase was followed by a measurement period of approximately 30 min where a total of three measurements were performed (BG, PL and OL). For treatment I and S the end expiratory concentration had to be adjusted before each equilibration phase. Jugular venous blood samples were taken for serum glucose, lactate and plasma concentrations of propofol and alfaxalone

In treatment I and S dogs were induced by mask with 5% isoflurane (Isofluran CP®, CP-Pharma) or 6% sevoflurane (SevoFlo®, EcuPhar GmbH) in 100% oxygen with a flow of 8 L min^− 1^. In treatment P and A, induction took place with 10 mg kg^− 1^ propofol (Narcofol®, 10 mg mL^− 1^, CP Pharma) or 3 mg kg^− 1^ alfaxalone (Alfaxan®, 10 mg mL^− 1^, Jurox Pty Ltd) injected over a period of 60 s. Isoflurane, sevoflurane, propofol and alfaxalone were administered in three different doses. At each dose, proton magnetic resonance spectroscopic measurements were performed. A 30 min equilibration period was allowed before each measurement was conducted. The order and doses for isoflurane, sevoflurane were 2.1 vol%/2.8 vol%/1.7 vol% and 3.5 vol%/4.7 vol%/2.8 vol%, respectively, representing 1.5 MAC, 2 MAC and 1.2 MAC (1 MAC_ISO_ = 1.39 vol% and 1 MAC_SEVO_ = 2.36 vol% according to T Kazama and K Ikeda [28], values above are calculated and rounded, respectively). For propofol and alfaxalone, doses were 30 mg kg^− 1^ h^− 1^, 45 mg kg^− 1^ h^− 1^, 60 mg kg^− 1^ h^− 1^ and 10 mg kg^− 1^ h^− 1^, 15 mg kg^− 1^ h^− 1^, 20 mg kg^− 1^ h^− 1^, respectively (Fig. 1). These anesthetics were administered as an incremental increase in dose rate to try and avoid period to period interaction with the possible accumulation of the injectable anesthetics.

After induction, intubation and instrumentation the animals were placed in sternal recumbency in the MRI scanner and the first equilibration phase started (in treatment I and S additional time was needed to adjust the end-expiratory concentration before each equilibration phase). Volume-controlled intermittent positive pressure ventilation was adjusted to maintain end tidal CO_2_ in the eucapnic range of 35 and 45 mmHg. Measurements included pulse rate, SpO_2_, P_E_’CO_2_, respiratory rate, inspiratory and expiratory isoflurane-, sevoflurane- and oxygen concentrations and body temperature.

For cardiovascular support a buffered balanced electrolyte solution (Sterofundin®) and 6% HES 130/0.42 (Venofundin® 6% HES) at a rate of 5 mL kg^− 1^ h^− 1^ dobutamine 3 μg kg^− 1^ min^− 1^ was administered to support cardiac output. Infusion rates of Sterofundin® were reduced with increasing infusion volume of injectable anesthetics, respectively.

The dogs were recovered in a quiet environment and warmed until physiologic body temperature was reached. They were returned into their group on the following day.

### Blood sampling

Blood samples for plasma lactate, plasma triglycerides, blood gases and plasma glucose were taken after each measurement period via the central venous catheter. In Treatment P and A, additional blood was taken for plasma concentrations of propofol and alfaxalone at the end of each measurement period before increasing the dose rate (Fig. 1). Plasma glucose concentration (Reflovet® Plus – Roche; reflexionphotometric measurement) and blood gases (ABL80 FLEX – Radiometer GMBH; amperometric/potentiometric measurement) were measured immediately whereas blood samples for plasma lactate, plasma triglycerides and plasma concentrations of propofol and alfaxalone were centrifuged at 11,800 rpm for 2 min, plasma was separated and immediately stored at − 82 °C until analyzed. Plasma lactate and plasma triglycerides were analyzed via absorption photometric measurement (cobas c311 – Roche Hitachi). Propofol plasma concentrations were quantified by means of gas chromatography - mass spectrometry (GC/MS) after solid-phase extraction and alfaxalone plasma concentrations were determined with liquid chromatography – tandem mass spectrometry (LC/MS) after solid phase extraction [29].

### Imaging protocol

Magnetic resonance imaging was performed with a 3.0 T Philips Achieva MRI scanner (Philips MedicalSystems, Best, the Netherlands) with a 8-channel receive-transmit knee coil (Philips MedicalSystems, Best, the Netherlands). For morphological imaging of the brain, transverse, dorsal, and sagittal turbo spin echo T2-weighted images were performed. Scan parameters were as follow: echo time, 80 milliseconds; repetition time, 3000 milliseconds; turbo spin factor, 15; matrix, 256 × 204; slice thickness, 3 mm; number of signal averages, 1. These images were performed during the first 30 min equilibration phase to reduce total time of anesthesia.

**Fig. 2:**
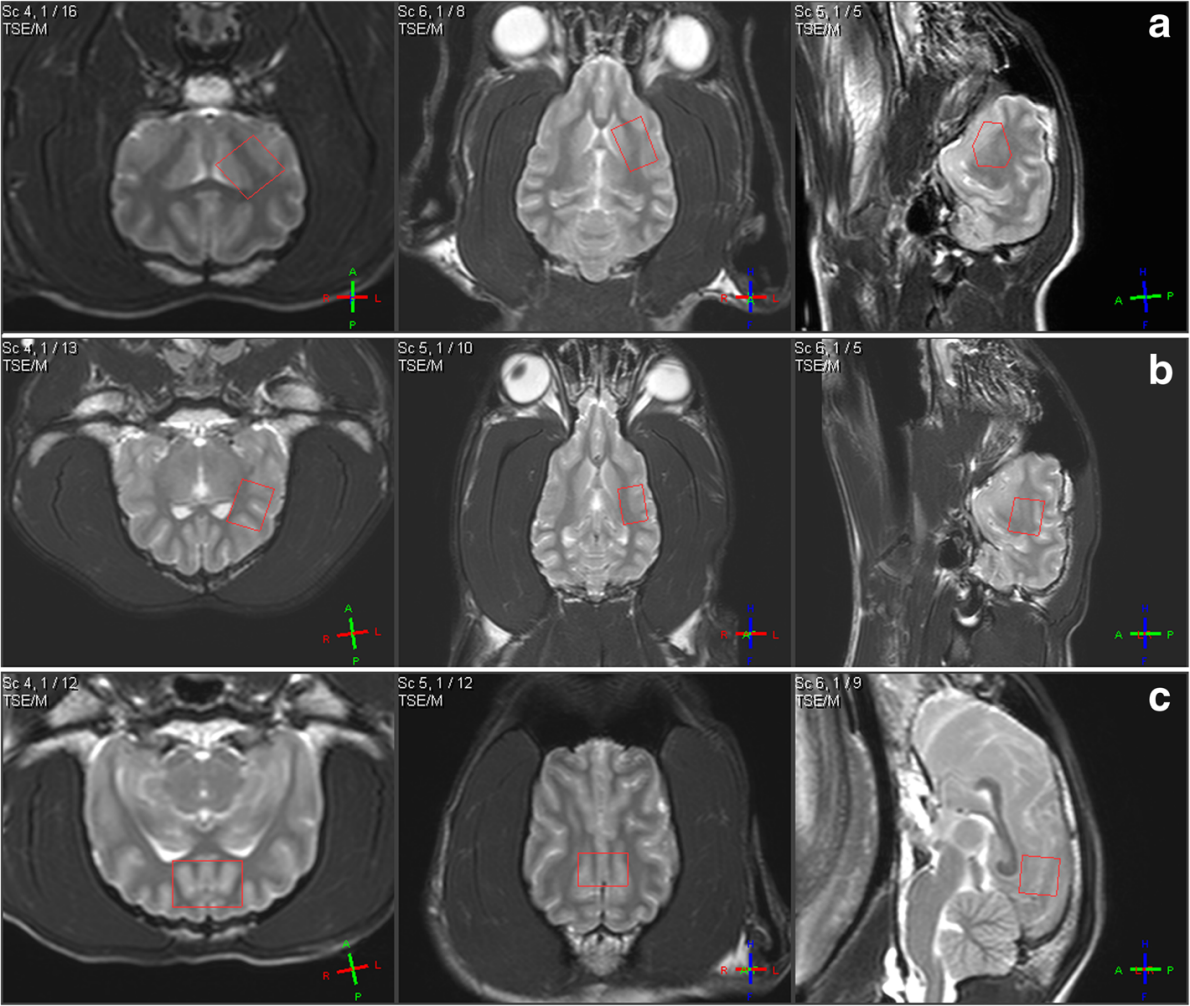
T2 weighted images of the brain of one beagle dog. Transversal (left), dorsal (middle), parasagittal (right; **a** and **b**) or sagittal (right; **c**) view; The red rectangles display the ^1^H MRS voxels of interests (VOI) for left basal ganglia (**a**), left parietal lobe (**b**) and midline occipital lobe (**c**)

Single voxel ^1^H MRS was performed by one of the investigators (FS) by use of short-echo time point resolved technique (PRESS) with voxels graphically prescribed from the T2-weighted images. Volume samples were performed over various regions of the brain parenchyma, including left basal ganglia (voxel of interest 1.5 cm^3^), left parietal lobe (voxel of interest 1.95 cm^3^), and midline occipital lobe (voxel of interest 1.5 cm^3^) (Fig. 2). Only the left hemisphere was analyzed, based on a previously reported study, in which differences in the metabolite concentration between left and right hemispheres were not found in the canine brain [15]. Parameters for the ^1^H MRS technique were as follow: echo time, shortest possible (32–34 milliseconds – usually 32 milliseconds in occipital lobe and 33 milliseconds in basal ganglia and parietal lobe); repetition time, 2000 milliseconds; number of sample averages, 256; bandwidth, 2000 Hz. Care was taken to avoid cerebrospinal fluid and peripheral soft and bony tissues to prevent lipid contamination. Before each ^1^H MRS acquisition, field homogeneity was optimized with a second-order automatic pencil-beam shim, which was followed by water suppression techniques (excitation). A water-unsuppressed image was also acquired to serve as a concentration reference for quantifying metabolite concentrations. In addition to a typical shim time of 2 min, each examination was acquired in 8 min and 36 s. Rejection criteria for the ^1^H MRS data were presence of an unstable baseline, line width > 10 Hz, signal-to-noise ratio < 6, presence of artifacts, or presence of lipid contamination.

### Data processing

Metabolite concentrations were estimated with an automated data processing spectral fitting (linear combination model) algorithm (LCModel). The software automatically adjusted the phase and chemical shift of the spectra, estimated the baseline, and performed eddy current correction. Relative metabolite concentrations and their uncertainties were estimated by fitting the spectrum to a basis set of spectra acquired from individual metabolites in solution. Seventeen metabolites were included in the linear combination model (alanine, aspartate, glucose, creatine, phosphocreatine, glutamine, glutamate, glycerophosphocholine, phosphocholine, lactate, lipids, myoinositol, N-acetylaspartate, *N*-acetylaspartylglutamate, scylloinositol, glutathione, and taurine). Only those metabolites with CRLBs < 20% were evaluated in this study.

One investigator (FS) reviewed MRI and ^1^H MRS images. The MRI images were assessed for abnormalities such as changes in signal intensity of the gray and white matter, presence of space-occupying lesions, mass effect, or ventriculomegaly. Metabolite concentrations relative to water and metabolite ratios with creatine as a reference were calculated from the ^1^H MRS data.

#### Statistical analysis

No comparable study on cerebral has been performed in dogs, so that a priori power analysis for calculation of sample size was based on the effect size from Jacob et al. 2012 performed in humans using a power of 90% and an alpha of 0.05.

Data were analyzed with aid of statistical software SAS Enterprise Guide (Version 6.1 M1, for Windows, SAS Institute Inc., Cary, NC, USA). Kolmogorov-Smirnov Test and Q-Q plots were used to test distribution of the data. Effect on cerebral metabolite concentrations were analyzed using a repeated measure ANOVA. This was performed for metabolite concentration relative to brain water content itself as well as for calculated metabolite ratios with creatine as internal reference metabolite.

Pearson’s correlations were performed to investigate the possible effect of plasma concentrations on metabolite concentrations and the effect of plasma sodium concentration on total NAA and inositol. Arithmetic mean of metabolite concentrations of the three doses for each anesthetic was calculated followed by a repeated measure ANOVA for determination of anesthetic effect. For differences of cerebral metabolite concentrations between investigated brain regions a repeated measures ANOVA for first measurement (equivalent to the second dose) in treatment S was performed. Wilcoxon Signed Rank Test was used for pulse rate, body temperature, plasma levels of lactate, glucose and triglycerides blood gas values as well as plasma drug concentrations. Bonferroni alpha adjustment for correction of alpha-error accumulation was applied when appropriate. Level of significance was set at alpha = 5%. Data are presented as mean ± standard deviation (SD).

## Results

**Fig. 3:**
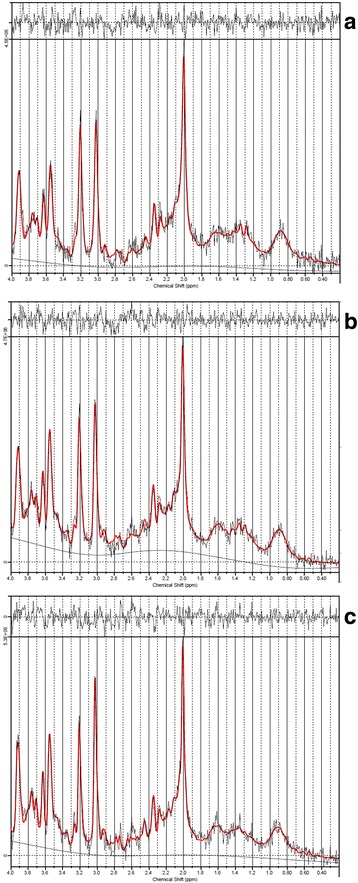
Example ^1^HMRS spectra of basal ganglia (**a**) parietal lobe (**b**) and occipital lobe (**c**) in one dog receiving 1.5 MAC (2.1 vol%) isoflurane. The x-axis represents the chemical shift in ppm of each metabolite and the y-axis represents the signal intensity

The T2-weighted images revealed no morphological abnormalities. Out of 360 planned measurements (10 dogs × 4 treatments × 3 doses × 3 brain regions) 348 spectra were obtained. Inadequate anesthesia led to abortion of trial in three dogs (two dogs receiving isoflurane 1.7 vol% (1.2 MAC) and in one dog receiving propofol only measurements at a rate of 45 mg kg^− 1^ h^− 1^ could be performed). Four spectroscopic measurements were discarded from isoflurane treatment due to insufficient signal-to-noise ratio (BG 2.8 vol% & 1.7 vol% and OL 2.1 vol% & 2.8 vol% - 4 measurements). In total, 344 spectra were included in the study. All spectra included were of good quality with a signal-to-noise-ratio (mean ± SD) of 14.07 ± 5.66 and linewidths (mean ± SD) of 4.75 ± 2.37 Hz. Example spectra of all three brain regions of one dog undergoing treatment I are shown in Fig. 3.

Changes in metabolite concentration relative to brain water content between the investigated brain regions followed the same trend with all anesthetics and are exemplary listed for the first measurement (equivalent to second Dose) in treatment S only (mean ± SD displayed in Tables 1, 2, 3, 4 and 5). Creatine concentration differed between all three brain regions. The lowest concentration in parietal lobe differed to the highest in occipital lobe (*p* < 0.0001) and to basal ganglia (*p* = 0.0001). Occipital lobe differed also to basal ganglia (*p* = 0.00156). Glx was lower in parietal lobe compared to basal ganglia (*p* = 0.0045) and occipital lobe (*p* = 0.041). N-acetylaspartate is lowest in basal ganglia being significant to the highest concentration in occipital lobe (*p* = 0.0445). The ratio to creatine was higher in parietal lobe compared to basal ganglia (*p* < 0.0001) and occipital lobe (*p* < 0.0001). Total choline was higher in in basal ganglia compared to parietal (*p* < 0.0001) and occipital lobes (*p* < 0.0001) but the ratio in occipital lobe was lowest compared to basal ganglia (*p* < 0.0001) and parietal lobe (*p* < 0.0001). Myoinositol ratios to creatine were higher in parietal lobe compared to basal ganglia (*p* = 0.0064) and occipital lobe (*p* = 0.0002).

**Table 1:** Mean ± SD of Glx concentrations (mmol L-1 of brain water) and Glx/tCr ratios

			Dose 1	Dose 2	Dose 3	Arithmetic mean
			Mean ± SD	Mean ± SD	Mean ± SD	Mean ± SD
GLX	BG	Isoflurane	13.568 ± 1.196	13.017 ± 1.037	13.039 ± 1.703	13.146 ± 1.003
		Sevoflurane	12.532 ± 1.369	12.931 ± 1.156	13.226 ± 1.266	12.896 ± 1.005
		Propofol	12.388 ± 1.066	12.730 ± 1.191	12.839 ± 1.582	12.760 ± 1.105
		Alfaxalone	12.941 ± 1.353	13.133 ± 1.670	13.423 ± 1.756	13.166 ± 1.233
	PL	Isoflurane	11.831 ± 2.167^AB^	13.119 ± 1.695^A^	11.821 ± 1.911^B^	12.345 ± 1.674^1^
		Sevoflurane	11.189 ± 1.375	10.632 ± 1.301	10.767 ± 0.888	10.863 ± 0.997^12^
		Propofol	10.201 ± 1.818	10.165 ± 1.669	10.481 ± 2.286	10.295 ± 1.722^2^
		Alfaxalone	10.087 ± 2.052	10.766 ± 2.084	10.315 ± 1.418	10.390 ± 1.353^2^
	OL	Isoflurane	12.957 ± 1.341	12.263 ± 1.648	13.083 ± 1.845	12.913 ± 1.463
		Sevoflurane	12.869 ± 2.277	12.311 ± 1.752	12.700 ± 1.539	12.627 ± 1.694
		Propofol	12.043 ± 0.400	12.135 ± 0.809	12.587 ± 1.289	12.223 ± 0.646
		Alfaxalone	12.555 ± 1.586	12.320 ± 1.075	12.609 ± 1.055	12.495 ± 1.032
GLX/tCr	BG	Isoflurane	2.326 ± 0.136	2.118 ± 0.144	2.215 ± 0.416	2.196 ± 0.214
		Sevoflurane	2.082 ± 0.221	2.032 ± 0.185	2.117 ± 0.180	2.077 ± 0.127
		Propofol	2.082 ± 0.199	2.109 ± 0.153	2.136 ± 0.216	2.119 ± 0.141
		Alfaxalone	2.045 ± 0.182	2.082 ± 0.232	2.091 ± 0.290	2.073 ± 0.175
	PL	Isoflurane	2.177 ± 0.365	2.353 ± 0.352	2.110 ± 0.342	2.224 ± 0.306
		Sevoflurane	2.064 ± 0.265	1.908 ± 0.240	1.932 ± 0.178	1.968 ± 0.197
		Propofol	1.826 ± 0.385	1.858 ± 0.388	1.931 ± 0.516	1.865 ± 0.396
		Alfaxalone	1.857 ± 0.365	2.001 ± 0.451	1.915 ± 0.242	1.924 ± 0.272
	OL	Isoflurane	1.964 ± 0.302	1.800 ± 0.243	2.028 ± 0.278	1.984 ± 0.287
		Sevoflurane	1.914 ± 0.353	1.810 ± 0.300	1.870 ± 0.240	1.864 ± 0.282
		Propofol	1.777 ± 0.063	1.838 ± 0.147	1.834 ± 0.156	1.839 ± 0.131
		Alfaxalone	1.868 ± 0.280	1.839 ± 0.213	1.922 ± 0.193	1.877 ± 0.220

Mean ± SD for the three different doses and the arithmetic mean of the three doses for isoflurane, sevoflurane, propofol and alfaxalone in basal ganglia (BG), parietal lobe (PL) and occipital lobe (OL). Different alphabetical superscripts differ significantly (*p* < 0.05) between different doses of each anesthetic and different numerical superscripts differ significantly between arithmetic mean Glx concentrations of each anesthetic. For treatment I and S: Dose 1 ≙ time 3; Dose 2 ≙ time 1; Dose 3 ≙ time 2

**Table 2:** Mean ± SD of tNAA concentrations (mmol L-1 of brain water) and tNAA/tCr ratios

			Dose 1	Dose 2	Dose 3	Arithmetic mean
			Mean ± SD	Mean ± SD	Mean ± SD	Mean ± SD
tNAA	BG	Isoflurane	6.911 ± 0.753	7.318 ± 0.425	6.955 ± 0.387	7.094 ± 0.339
		Sevoflurane	7.100 ± 0.548	7.297 ± 0.554	7.219 ± 0.512	7.205 ± 0.509
		Propofol	7.288 ± 0.484^AB^	7.007 ± 0.466^A^	7.524 ± 0.618^B^	7.226 ± 0.444
		Alfaxalone	7.104 ± 0.472^A^	7.499 ± 0.410^AB^	7.724 ± 0.541^B^	7.442 ± 0.376
	PL	Isoflurane	7.485 ± 0.585	7.692 ± 0.379	7.575 ± 0.360	7.583 ± 0.321
		Sevoflurane	7.664 ± 0.490	7.631 ± 0.690	7.673 ± 0.526	7.656 ± 0.540
		Propofol	7.622 ± 0.538	7.592 ± 0.617	7.667 ± 0.528	7.595 ± 0.516
		Alfaxalone	7.681 ± 0.364	7.704 ± 0.376	7.805 ± 0.449	7.730 ± 0.282
	OL	Isoflurane	7.725 ± 0.309	7.898 ± 0.497	7.599 ± 0.934	7.696 ± 0.556
		Sevoflurane	7.788 ± 0.642	7.907 ± 0.496	7.837 ± 0.492	7.844 ± 0.494
		Propofol	7.859 ± 0.393	7.741 ± 0.490	7.628 ± 0.398	7.687 ± 0.432
		Alfaxalone	7.680 ± 0.472	7.675 ± 0.559	7.426 ± 0.724	7.594 ± 0.498
tNAA/tCr	BG	Isoflurane	1.187 ± 0.126	1.195 ± 0.095	1.212 ± 0.108	1.203 ± 0.095
		Sevoflurane	1.183 ± 0.125	1.148 ± 0.108	1.157 ± 0.086	1.163 ± 0.099
		Propofol	1.229 ± 0.100	1.166 ± 0.117	1.255 ± 0.102	1.205 ± 0.107
		Alfaxalone	1.126 ± 0.098	1.272 ± 0.238	1.202 ± 0.105	1.200 ± 0.110
	PL	Isoflurane	1.381 ± 0.100	1.377 ± 0.103	1.349 ± 0.071	1.365 ± 0.065
		Sevoflurane	1.416 ± 0.134	1.369 ± 0.108	1.381 ± 0.083	1.389 ± 0.095
		Propofol	1.361 ± 0.151	1.382 ± 0.172	1.404 ± 0.174	1.370 ± 0.159
		Alfaxalone	1.419 ± 0.135	1.429 ± 0.134	1.450 ± 0.130	1.433 ± 0.119
	OL	Isoflurane	1.167 ± 0.078	1.158 ± 0.047	1.170 ± 0.053	1.175 ± 0.066
		Sevoflurane	1.157 ± 0.093	1.158 ± 0.052	1.153 ± 0.069	1.156 ± 0.063
		Propofol	1.160 ± 0.054^AB^	1.171 ± 0.060^A^	1.113 ± 0.052^B^	1.156 ± 0.054
		Alfaxalone	1.137 ± 0.076	1.144 ± 0.089	1.127 ± 0.064	1.136 ± 0.064

Mean ± SD for the three different doses and the arithmetic mean of the three doses for isoflurane, sevoflurane, propofol and alfaxalone in basal ganglia (BG), parietal lobe (PL) and occipital lobe (OL). Different alphabetical superscripts differ significantly (*p* < 0.05) between different doses of each anesthetic

**Table 3:** Mean ± SD of tCh concentrations (mmol L-1 of brain water) and tCh/tCr ratios

			Dose 1	Dose 2	Dose 3	Arithmetic mean
			Mean ± SD	Mean ± SD	Mean ± SD	Mean ± SD
tCh	BG	Isoflurane	1.887 ± 0.112^A^	2.079 ± 0.110	1.932 ± 0.166^A^	1.969 ± 0.089^1^
		Sevoflurane	1.971 ± 0.109^A^	2.107 ± 0.145^B^	2.068 ± 0.128^AB^	2.049 ± 0.103^12^
		Propofol	2.034 ± 0.118	2.015 ± 0.109	2.010 ± 0.139	2.026 ± 0.090^12^
		Alfaxalone	2.069 ± 0.103	2.082 ± 0.105	2.108 ± 0.123	2.086 ± 0.080^2^
	PL	Isoflurane	1.805 ± 0.180^A^	1.885 ± 0.129^B^	1.841 ± 0.132^AB^	1.843 ± 0.134
		Sevoflurane	1.721 ± 0.107^A^	1.806 ± 0.100^B^	1.747 ± 0.133^AB^	1.758 ± 0.101
		Propofol	1.843 ± 0.117	1.773 ± 0.233	1.788 ± 0.204	1.768 ± 0.218
		Alfaxalone	1.854 ± 0.103	1.822 ± 0.107	1.814 ± 0.162	1.830 ± 0.112
	OL	Isoflurane	1.616 ± 0.288	1.805 ± 0.121	1.667 ± 0.202	1.653 ± 0.258^1^
		Sevoflurane	1.707 ± 0.148^A^	1.807 ± 0.094^B^	1.768 ± 0.132^AB^	1.761 ± 0.118^12^
		Propofol	1.795 ± 0.122	1.831 ± 0.122	1.832 ± 0.116	1.823 ± 0.105^2^
		Alfaxalone	1.812 ± 0.140	1.802 ± 0.102	1.789 ± 0.092	1.801 ± 0.097^12^
tCh/tCr	BG	Isoflurane	0.324 ± 0.021	0.339 ± 0.017	0.331 ± 0.043	0.330 ± 0.027
		Sevoflurane	0.329 ± 0.033	0.332 ± 0.031	0.331 ± 0.020	0.330 ± 0.025
		Propofol	0.342 ± 0.017	0.335 ± 0.025	0.335 ± 0.024	0.337 ± 0.020
		Alfaxalone	0.328 ± 0.020	0.331 ± 0.018	0.328 ± 0.017	0.329 ± 0.016
	PL	Isoflurane	0.334 ± 0.039	0.338 ± 0.030	0.329 ± 0.029	0.332 ± 0.030
		Sevoflurane	0.318 ± 0.033	0.324 ± 0.021	0.313 ± 0.023	0.319 ± 0.022
		Propofol	0.329 ± 0.031	0.322 ± 0.047	0.325 ± 0.028	0.318 ± 0.043
		Alfaxalone	0.342 ± 0.032	0.337 ± 0.027	0.338 ± 0.040	0.339 ± 0.030
	OL	Isoflurane	0.242 ± 0.031^A^	0.265 ± 0.010^B^	0.257 ± 0.008^AB^	0.251 ± 0.026
		Sevoflurane	0.253 ± 0.020	0.265 ± 0.015	0.260 ± 0.023	0.260 ± 0.018
		Propofol	0.265 ± 0.014	0.278 ± 0.028	0.268 ± 0.023	0.275 ± 0.027
		Alfaxalone	0.268 ± 0.018	0.268 ± 0.016	0.273 ± 0.019	0.270 ± 0.015

Mean ± SD for the three different doses and the arithmetic mean of the three doses for isoflurane, sevoflurane, propofol and alfaxalone in basal ganglia (BG), parietal lobe (PL) and occipital lobe (OL). Different alphabetical superscripts differ significantly (*p* < 0.05) between different doses of each anesthetic different numerical superscripts differ significantly between arithmetic mean tCh concentrations of each anesthetic

**Table 4:** Mean ± SD of Ins concentrations (mmol/L of brain water) and Ins/tCr ratios

			Dose 1	Dose 2	Dose 3	Arithmetic mean
			Mean ± SD	Mean ± SD	Mean ± SD	Mean ± SD
Ins	BG	Isoflurane	7.931 ± 0.343	7.983 ± 0.589	7.730 ± 0.824	7.876 ± 0.529
		Sevoflurane	8.365 ± 0.816	8.470 ± 0.780	8.344 ± 0.710	8.393 ± 0.701
		Propofol	8.033 ± 0.561	8.056 ± 0.778	8.109 ± 0.646	8.100 ± 0.601
		Alfaxalone	8.325 ± 0.606	8.446 ± 0.689	8.605 ± 0.487	8.459 ± 0.470
	PL	Isoflurane	7.679 ± 0.996	8.176 ± 0.861	8.070 ± 0.716	8.016 ± 0.835
		Sevoflurane	8.165 ± 0.445	8.367 ± 0.528	8.131 ± 0.485	8.221 ± 0.414
		Propofol	8.178 ± 1.075	7.976 ± 1.266	7.833 ± 1.439	7.992 ± 1.196
		Alfaxalone	7.967 ± 1.103	8.177 ± 1.078	8.188 ± 0.888	8.111 ± 0.954
	OL	Isoflurane	8.005 ± 0.885	8.233 ± 0.871	8.077 ± 1.171	8.016 ± 1.087
		Sevoflurane	8.362 ± 0.620	8.588 ± 0.554	8.342 ± 0.677	8.431 ± 0.579
		Propofol	8.382 ± 0.611	8.455 ± 0.749	8.336 ± 0.593	8.501 ± 0.752
		Alfaxalone	8.524 ± 0.649	8.449 ± 0.629	8.523 ± 0.572	8.499 ± 0.591
Ins/tCr	BG	Isoflurane	1.365 ± 0.114	1.299 ± 0.082	1.331 ± 0.100	1.326 ± 0.075
		Sevoflurane	1.389 ± 0.132	1.333 ± 0.137	1.336 ± 0.095	1.353 ± 0.103
		Propofol	1.351 ± 0.124	1.335 ± 0.112	1.353 ± 0.114	1.307 ± 0.167
		Alfaxalone	1.319 ± 0.108	1.334 ± 0.099	1.340 ± 0.110	1.331 ± 0.081
	PL	Isoflurane	1.417 ± 0.173	1.462 ± 0.145	1.439 ± 0.112	1.443 ± 0.136
		Sevoflurane	1.510 ± 0.135	1.502 ± 0.084	1.457 ± 0.121	1.490 ± 0.107
		Propofol	1.459 ± 0.221	1.451 ± 0.266	1.431 ± 0.271	1.440 ± 0.242
		Alfaxalone	1.462 ± 0.214	1.509 ± 0.215	1.525 ± 0.211	1.498 ± 0.200
	OL	Isoflurane	1.203 ± 0.081	1.205 ± 0.092	1.245 ± 0.113	1.220 ± 0.103
		Sevoflurane	1.242 ± 0.090	1.258 ± 0.077	1.228 ± 0.115	1.243 ± 0.089
		Propofol	1.235 ± 0.056	1.287 ± 0.197	1.120 ± 0.326	1.256 ± 0.232
		Alfaxalone	1.262 ± 0.098	1.259 ± 0.100	1.304 ± 0.160	1.275 ± 0.113

Mean ± SD for the three different doses and the arithmetic mean of the three doses for isoflurane, sevoflurane, propofol and alfaxalone in basal ganglia (BG), parietal lobe (PL) and occipital lobe (OL).

**Table 5:** Mean ± SD of tCr concentrations (mmol L-1 of brain water)

			Dose 1	Dose 2	Dose 3	Arithmetic mean
			Mean ± SD	Mean ± SD	Mean ± SD	Mean ± SD
tCr	BG	Isoflurane	5.857 ± 0.647	6.154 ± 0.400	5.699 ± 0.657	5.925 ± 0.498^1^
		Sevoflurane	6.038 ± 0.486^A^	6.371 ± 0.338^B^	6.249 ± 0.353^AB^	6.219 ± 0.344^12^
		Propofol	5.958 ± 0.230	6.043 ± 0.496	6.011 ± 0.451	6.026 ± 0.328^12^
		Alfaxalone	6.320 ± 0.218	6.310 ± 0.439	6.446 ± 0.429	6.359 ± 0.283^2^
	PL	Isoflurane	5.419 ± 0.180	5.598 ± 0.303	5.607 ± 0.210	5.556 ± 0.200
		Sevoflurane	5.441 ± 0.460	5.580 ± 0.383	5.584 ± 0.304	5.535 ± 0.347
		Propofol	5.627 ± 0.312	5.531 ± 0.407	5.494 ± 0.394	5.576 ± 0.342
		Alfaxalone	5.439 ± 0.350	5.420 ± 0.358	5.402 ± 0.491	5.420 ± 0.376
	OL	Isoflurane	6.647 ± 0.482	6.825 ± 0.371	6.496 ± 0.758	6.566 ± 0.527
		Sevoflurane	6.734 ± 0.176	6.831 ± 0.319	6.809 ± 0.403	6.792 ± 0.250
		Propofol	6.783 ± 0.321	6.624 ± 0.456	6.862 ± 0.355	6.670 ± 0.487
		Alfaxalone	6.761 ± 0.268	6.718 ± 0.249	6.588 ± 0.559	6.689 ± 0.325

Mean ± SD for the three different doses and the arithmetic mean of the three doses for isoflurane, sevoflurane, propofol and alfaxalone in basal ganglia (BG), parietal lobe (PL) and occipital lobe (OL). Different alphabetical superscripts differ significantly (*p* < 0.05) between different doses of each anesthetic different numerical superscripts differ significantly between arithmetic mean tCr concentrations of each anesthetic

A lactate signal was not detected at any time. Few changes in cerebral metabolite concentrations relative to brain water content were found among the doses of the four anesthetics. Significant differences were detected for Glx in treatment I in parietal lobe but these changes did not coincide with either time or dose (Table 1). The total NAA (tNAA) concentration relative to water content in basal ganglia increased in a dose- or time-dependent manner in treatment A to reach a significant level between the first and third dose. Significant differences in tNAA were detected in treatment P in basal ganglia but these changes did not coincide with either time or dose (Table 2). Total choline concentration relative to brain water content decreased over time with both volatile anesthetics being significant in all brain regions except for treatment I in the occipital lobe (Table 3). Cerebral concentration of inositol revealed no significant differences (Table 4). Creatine concentration relative to cerebral water decreased with treatment S time-dependently (Table 5).

Metabolite ratios were calculated with creatine as internal reference metabolite. Total NAA to creatine ratio in treatment P in the occipital lobe showed significant differences between doses without relationship to dose or time (Table 2). A reduction in total choline to creatine ratio over time was detected for treatment I in the occipital lobe only (Table 3).

For comparison of the four anesthetics metabolite concentrations and their ratios with creatine as the denominator were arithmetically averaged over the three doses of each anesthetic. In treatment I higher levels of Glx were detected compared to injectable anesthetics in the parietal lobe (Table 1). Total choline was lower with isoflurane compared to treatment A in the basal ganglia and treatment P in the occipital lobe (Table 3) and creatine was lower with isoflurane compared to treatment A in the basal ganglia (Table S5)**.** Metabolite ratios to creatine revealed no significant differences between anesthetics (Tables 1, 2, 3, 4 and 5).

**Fig. 4:**
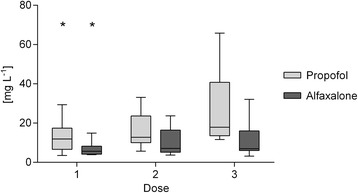
Plasma concentration of propofol and alfaxalone in mg L^− 1^ for doses 1, 2 and 3. First and third quartiles are defined by the boxes and the median by the band inside. Minimum and maximum is indicated by the whiskers. * Significant difference to dose 2 and 3 (*p* < 0.05)

Plasma concentrations of alfaxalone and propofol showed a wide individual range. Mean plasma concentrations increased with infusion rate with a statistically significant differentiation between the low dose and the middle and high dose, but not between the middle and the high dose for both injectable anesthetics (Fig. 4). Pearson’s correlation asserted no interdependence of plasma concentration of propofol and alfaxalone on metabolite concentration and of sodium levels on total NAA and inositol.

In Treatment P, blood pH, HCO_3_^−^ and sBE decreased. Plasma levels of glucose showed a mild increase in Treatment I but stayed within clinical reference. Plasma lactate increased dose-dependently with both volatile anesthetics and plasma triglycerides increased with administration of propofol (Table 6).

**Table 6:** pH, HCO3-, sBE plasma glucose, −lactate and –triglycerides

		Baseline	Measurement 1	Measurement 2	Measurement 3
		Mean ± SD	Mean ± SD	Mean ± SD	Mean ± SD
pH	Isoflurane	7.38 ± 0.04^A^	7.35 ± 0.04	7.37 ± 0.05	7.38 ± 0.02^AB^
	Sevoflurane	7.39 ± 0.02^AB^	7.35 ± 0.02	7.36 ± 0.02	7.39 ± 0.04^A^
	Propofol	7.41 ± 0.02^B^	7.33 ± 0.03*	7.33 ± 0.04*	7.30 ± 0.04^*B^
	Alfaxalone	7.39 ± 0.05^AB^	7.34 ± 0.03	7.35 ± 0.03	7.35 ± 0.03^AB^
HCO_3_^−^ [mmol L^− 1^]	Isoflurane	21.01 ± 1.73^A^	20.44 ± 1.74^AB^	20.00 ± 1.37^AB^	21.00 ± 1.57^AB^
	Sevoflurane	22.22 ± 1.46^AB^	20.32 ± 1.09*^A^	20.86 ± 0.99^AB^	20.66 ± 1.24^AB^
	Propofol	22.01 ± 1.87^B^	20.10 ± 1.31*^A^	19.67 ± 1.34*^A^	18.58 ± 1.11*^A^
	Alfaxalone	21.71 ± 1.59^AB^	21.73 ± 1.07^B^	22.00 ± 1.04^B^	21.94 ± 0.92^B^
sBE [mmol L^− 1^]	Isoflurane	−2.77 ± 1.31^A^	−4.27 ± 1.95^AB^	−4.38 ± 1.85^AB^	−3.30 ± 1.59^AB^
	Sevoflurane	−2.02 ± 1.54^AB^	−4.30 ± 1.08*^AB^	−3.70 ± 1.09^AB^	−3.56 ± 1.16*^B^
	Propofol	− 1.84 ± 1.70^B^	− 4.86 ± 1.46*^A^	−5.27 ± 1.55*^A^	−6.67 ± 1.41*^A^
	Alfaxalone	−2.5 ± 1.33^AB^	− 3.08 ± 1.06^B^	−2.78 ± 1.05^B^	−2.85 ± 0.98^B^
Na^+^ [mmol L^− 1^]	Isoflurane	148.2 ± 1.48	147.7 ± 1^A^	146.8 ± 1*^B^	147.25 ± 1.2^AB^
	Sevoflurane	147.9 ± 1.66	148.1 ± 1.8	146.2 ± 2.2*^A^	146.2 ± 1.6*^A^
	Propofol	147.2 ± 1.48	147.33 ± 1.7^A^	147.6 ± 1.8^A^	148.89 ± 1.6*
	Alfaxalone	147.67 ± 1.66	149 ± 1.1*	150.7 ± 3.1*	149.8 ± 0.9*
Glucose [mmol L^− 1^]	Isoflurane	5.29 ± 0.25	5.84 ± 0.61^B^	6.13 ± 0.65^A^	6.00 ± 0.49*^ABC^
	Sevoflurane	5.29 ± 0.37	5.19 ± 0.21^AC^	5.56 ± 0.40^A^	5.59 ± 0.42^A^
	Propofol	5.20 ± 0.28	4.98 ± 0.38^A^	4.97 ± 0.27	4.92 ± 0.30^B^
	Alfaxalone	5.65 ± 0.63	5.83 ± 0.49^BC^	6.17 ± 0.93^A^	6.31 ± 0.66^C^
Lactate [mmol L^− 1^]	Isoflurane	1.06 ± 0.40^A^	2.03 ± 0.94*^A^	2.10 ± 0.69*^A^	1.97 ± 0.78
	Sevoflurane	1.01 ± 0.30^AB^	1.61 ± 0.56^AB^	1.89 ± 0.74*^AB^	1.58 ± 0.77
	Propofol	1.01 ± 0.33^B^	1.12 ± 0.34^B^	1.31 ± 0.55^AB^	1.41 ± 0.23
	Alfaxalone	0.95 ± 0.49^AB^	0.86 ± 0.83^B^	1.03 ± 0.64^B^	1.14 ± 0.57
Triglycerides [mmol L^− 1^]	Isoflurane	0.53 ± 0.17^A^	0.36 ± 0.13*^AB^	0.29 ± 0.13*^A^	0.36 ± 0.24^AB^
	Sevoflurane	0.53 ± 0.14^AB^	0.36 ± 0.14^A^	0.29 ± 0.11*^A^	0.27 ± 0.09*
	Propofol	0.55 ± 0.12^B^	2.82 ± 1.02*	2.97 ± 1.21*	3.46 ± 1.07*^A^
	Alfaxalone	0.59 ± 0.16^AB^	0.22 ± 0.06^*B^	0.19 ± 0.07*^A^	0.20 ± 0.06*^B^

Mean ± SD of the results of blood gas analysis (pH, HCO3- [mmol L-1], sBE [mmol L-1]) and plasma glucose [mmol L-1], −lactate [mmol L-1] and -triglycerides [mmol L-1] before anaesthesia, after measurement period 1, 2 and 3; * *p* < 0.05 compared to baseline within one Treatment, Different alphabetical superscripts differ in between treatments at one measurementpoint (*p* < 0.05)

Body temperature decreased over time in treatments I, S and A. In treatment P, body temperature started to increase during second measurement to reach baseline values again after the third measurement (Table 7).

**Table 7:** pulse rate and body temperature

		pre induction	post induction	measurement 1	measurement 2	measurement 3
		Mean ± SD	Mean ± SD	Mean ± SD	Mean ± SD	Mean ± SD
pulse rate [bpm]	Isoflurane	92.40 ± 18.23	123.90 ± 24.92*^A^	113.13 ± 8.81^AB^	125.29 ± 14.15*^A^	131.45 ± 15.44*^AB^
	Sevoflurane	96.00 ± 15.00	123.90 ± 14.52*^A^	104.65 ± 13.83^AB^	116.08 ± 11.94*^A^	127.37 ± 18.57*^A^
	Propofol	87.20 ± 8.85	98.70 ± 26.96^A^	90.11 ± 22.37^A^	100.47 ± 21.31^A^	103.20 ± 19.75
	Alfaxalone	94.00 ± 7.24	163.20 ± 29.47*	115.68 ± 26.35^B^	155.55 ± 37.31*	149.94 ± 19.40*^B^
body temperature [°C]	Isoflurane	38.48 ± 0.28	38.18 ± 0.53	37.31 ± 0.57*	37.25 ± 0.67*^AB^	36.93 ± 0.60*^A^
	Sevoflurane	38.46 ± 0.21	38.07 ± 0.51	37.24 ± 0.32*	37.02 ± 0.38*^A^	36.83 ± 0.41*^A^
	Propofol	38.39 ± 0.27	38.08 ± 0.53	37.71 ± 0.45*	37.84 ± 0.46*^B^	38.20 ± 0.37
	Alfaxalone	38.52 ± 0.30	38.26 ± 0.36	37.38 ± 0.54*	37.22 ± 0.63*^AB^	37.03 ± 0.66*^A^

Mean ± SD pulse rate [bpm] and body temperature [°C] pre and post induction and averaged over measurement period 1, 2 and 3. * *p* < 0.05 significant to baseline (pre induction) within one treatment and different alphabetical superscripts differ significantly between treatments at the same timepoint (*p* < 0.05)

### Recovery from Anaesthesia

All dogs in Treatment I and S were extubated within 2–5 min after discontinuing anesthesia and recovery was uneventful. The first dog in treatment A and the first dog in treatment P developed myoclonia and reverse sneezing. Therefore, isoflurane was given for 30 min after discontinuing the constant rate infusion of alfaxalone or propofol to avoid myclonia. After discontinuation of isoflurane the recovery period was uneventful with time to extubation from 23 to 68 min.

## Discussion

Using ^1^H MRS at 3 T we found no evidence of increased intracerebral concentration of lactate with isoflurane, sevoflurane, propofol or alfaxalone in the canine brain. There were only minor differences between doses and anesthetics related to tNAA, Choline, Creatine, Inositol and Glx.

Cerebral lactate, formerly seen only as a waste product of anaerobe glycolysis is nowadays considered a beneficial metabolite serving as an energy substrate amongst others during hypoglycemia [10, 30]. Cerebral levels of lactate were increased upon exposure to volatile anesthetics in rodents in a reversible manner [11, 21, 22]. This effect was independent of blood lactate concentration. By performing a linear regression analysis of brain lactate measured by ^1^H MRS and blood lactate concentration no relationship was determined in rats [22]. Isoflurane was also shown to enhance neuroprotective effects of exogenously administered lactate in an experimental stroke model [10]. As isoflurane led to a 6-fold increase in cerebral lactate in mice, we expected to be able to detect a sufficient lactate resonance in canine brain at 3 T. However, at this field strength we were not able to quantify cerebral lactate. In comparison to our results the previously reported rodent studies [21, 22] were able to reliably detect lactate levels with a field strength of 9.4 T. Jacob et al. [24] published 2-fold higher cerebral lactate values in children with sevoflurane compared to propofol detected at 3 T. However, these data included metabolite concentrations with associated CRLB values of “80% or fewer in 80% of the scans” and thus are not reliable. Uncertainties in metabolite concentrations in LcModel are expressed in CRLBs and in general only values of 20% or less are of acceptable reliability [31]. Lactate resonances in ^1^H MRS are only detectable during neuronal stimulation [32] and in pathologic conditions such as neoplastic or inflammatory lesions [12, 14] also in canine brain [27]. Under these circumstances lactate can reliably be detected also at 3 T. In various species and with different measuring techniques it has been shown that halogenated volatile anesthetics increase cerebral levels of lactate [11, 21–23]. As we did not detect lactate under influence of isoflurane and sevoflurane administered at a dose of 2 MAC we can conclude that halogenated volatile anesthetics do not have an effect on cerebral levels of lactate in dogs. However, technical difficulties such as coupling effects of the lactate doublet [33] and overlapping resonances of lactate and lipids resonating at the same region of the spectrum at this field strength cannot be excluded.

Increased serum lactate with volatile anesthetics can be explained by increased lactate production or decreased hepatic lactate clearance. Volatile anesthetics dose dependently decrease blood pressure by venodilation and reduced myocardial contractility. This could potentially lead to reduced peripheral perfusion with an increase in lactate production and a reduction in hepatic blood flow, thereby, decreasing hepatic clearance. In contrast, desflurane and propofol were shown to even increase peripheral perfusion index in a clinical study in humans by means of a new generation pulse oximeter [34]. Measurement of cardiac output was not performed. In dogs, 1.85 MAC isoflurane tended to increase hepatic blood flow [35]. Various studies have shown effects of volatile anesthetics on mitochondria [36–39]. Interference of volatile anesthetics with mitochondrial oxidation of NADH to NAD and thus reduced oxidative phosphorylation resulting in a shift to lactate production was shown for rat liver and monkey kidney cells [40, 41]. Therefore, the observed increased serum lactate levels are more likely related to direct mitochondrial effects than malperfusion.

Differences in metabolite concentrations between different brain regions presented in this paper show a similar pattern as previously published for canine brain [15, 17] and were independent of the anesthetic used. In humans, comparison of brain metabolite concentrations measured with different scanners and techniques showed good agreement but absolute values differed [42]. Data from other institutions can only be used as reference but direct comparison of data acquired on scanners from other institutions is not recommended.

In basal ganglia tNAA was the only metabolite showing dose-dependent increase of 8% in dogs receiving alfaxalone, being significantly different (*p* = 0.0036) between the first and third dose. However, this increase might also be time associated as the dose in treatment P and A increased over time. In the parietal lobe a similar increase was detected but statistical significance was not reached. In the occipital lobe, no changes were noted. The detected increase in cerebral levels of tNAA is unexpected. In pathologic conditions associated with neuronal loss and dysfunction such as demyelinating and neurodegenerative disorders [43] tNAA is decreased. As we did not detect a decrease in tNAA there seems to be no neuronal degeneration or loss occurring during anesthesia. Increases in tNAA are rare and only described for canvan’s disease in humans. Canvan’s disease is an autosomal recessive leukodystrophy with absence of aspartoacylase resulting in lack of NAA-degradation [12] with increases in tNAA of about 50% or more [44, 45]. To the author’s knowledge, this condition has not been reported in dogs and does not explain the detected changes during anesthesia.

Increase in choline-containing-compounds with isoflurane was described for mice [21]. An increase in cerebral acetylcholine is described for general anesthesia in mice [46, 47]. As changes in cerebral acetylcholine have only little effect on total choline concentration, adrenergic pathways were suggested as the α2-agonist medetomidine revoked isoflurane induced increases in choline containing compounds in mice [21]. We detected a time-dependent reduction in total choline with volatile anesthetics (Table 3). Predominantly lower levels of total choline were also detected with volatile anesthetics when comparing the arithmetic mean of total choline concentration of the three doses of each anesthetic (Table 3). Lower levels of glycerophosphocholine and phosphocholine with isoflurane compared to propofol were also depicted in rats [22]. Another study in piglets also reported decreases in Cho [48] and hypothesized oxidative stress due to anesthesia affecting osmoregulation in astrocytes [49]. Isoflurane was shown to cause oxidative stress in human neuroglioma cells [50] whereas propofol had antioxidant properties in cultured astrocytes [51] explaining the different effects on choline levels.

Higher levels of Glx were present in in the parietal lobe with isoflurane compared to propofol and alfaxlaone and with both volatile anesthetics Glx was higher than with the injectable anesthetics in other investigated brain regions. Elevated cerebral concentrations of glutamate and glutamine might be mediated by effects of volatile anesthetics on cerebral glutamate transporters and thus interference in glutamate turnover [52, 53]. The changes described here for Glx are, however, not distinctive enough to allow a conclusive statement.

Total NAA and myo-Inositol have been described as cerebral osmolytes [54, 55]. Changes in plasma osmolarity and thus the administered cardiovascular support may have impact on these metabolites. Plasma osmolarity is largely dependant on plasma levels of sodium. Plasma levels of sodium stayed within reference limits but few differences were detected compared to baseline. Pearson’s correlation asserted no interdependence of sodium with total NAA and inositol. Also, lack of significant changes in cerebral concentration of these metabolites consistent in all treatments with increasing amount of fluids administered implies, that the chosen cardiovascular support did not affect these metabolites at measured times.

Changes in body temperature were similar for isoflurane, sevoflurane and alfaxalone showing a decrease over time due their effects on hypothalamic thermoregulation and decreased muscle tone and metabolic rate [56]. In dogs receiving propofol body temperature started to rise again after an initial decline with the high infusion rate. A link to propofol infusion syndrome (PRIS) [57], a contemporary issue in human medicine can be ruled out because rhabdomyolysis, bradycardia, hyperkalemia and other clinical signs of PRIS were not seen in these dogs and overall duration of propofol infusion was short. As the temperature response to high doses of propofol was the same in all dogs, a specific mechanism can be suspected but to the authors’ knowledge has not been described before. In conjunction with the decrease in HCO_3_^−^, sBE, pH and glucose, increased muscle activity induced by high dose propofol is a possible explanation but clinically no increase in muscle tone or muscle twitching was observed during the experiment. The high doses were chosen consciously to better reveal effects on brain metabolites. As these doses exceed clinically administered infusion rates by far, this effect on body temperature is not seen in clinical cases.

Turbidity of hyperlipemic plasma in propofol plasma samples might interfere with photometric measurements of lactate, glucose and triglycerides and might falsify results. However, the lipaemic index as an extinction based reference value did not exceed the critical lipaemic index values specified for the tests used.

Except for high dose of isoflurane and middle and high dose of alfaxalone serum glucose stayed within reference limits but showed slight changes in all anesthetics. Increase in serum glucose during anesthesia is well documented as a result of reduced insulin secretion during anesthesia with different volatile agents in dogs, swine and humans [58–62]. Propofol was shown to slightly increase insulin secretion in rhesus monkeys [63] leading to lower glucose levels. Alfaxalone was shown to have no effect on glucose and insulin concentration up to 15 min after bolus induction in acepromazine and hydromorphone premedicated dogs [64]. When administered in supraclinical doses over a long period, our data suggest a similar effect on glucose as with volatile anesthetics but insulin concentration was not determined.

Several limitations of this study can be specified concerning magnetic resonance protocol, post processing procedure and anesthetic management. Compared to human brain, complete differentiation of grey and white matter for proton magnetic resonance spectroscopy was not possible so that the fraction from grey and white matter in the voxel of interest in the same brain region differ in between measurements. Absolute metabolite concentrations were not determined as correction for T1 and T2 effects involves excessively prolonged image acquisition times.

Individual MAC and MIR were not determined. Main focus of this study was to evaluate dose dependent effects of the four investigated anesthetics on the brain. The MAC and MIR are primarily measures for anesthetic potency related to immobility in response to a maximal noxious stimulus [65, 66]. In contrast to unconsciousness, immobility is mainly mediated via spinal cord effects [67, 68], therefore individual MAC and MIR values were not considered to be necessary for comparing anesthetic effects on the brain.

The injectable anesthetics were administered at incrementally increasing dose rate to try and avoid a possible period effect due to accumulation of drug, particularly at supra-clinical dose rates. Plasma concentrations, on average, for both alfaxalone and propofol increased with increasing infusion rates but individual patient variability was observed, leading to some overlap between plasma concentrations at middle and high infusion rates.

To the authors knowledge this study was the first study evaluating effects of commonly used anesthetics on cerebral metabolism in different regions of the canine brain.

## Conclusion

Neither with isoflurane nor with sevoflurane elevated levels of cerebral lactate could be detected. For tNAA, choline, creatine, inositol and Glx only minor differences between treatments were seen. These differences seem to be without clinical relevance. The effect of other peri-anesthetically administered drugs like sedatives and analgesics on canine cerebral metabolites still need to be determined in further studies.

## Abbreviations

^1^H MRS: Proton magnetic resonance spectroscopy
ANOVA: Analysis of variance
ASA: American Society of Anesthesiologists
Cho: Choline
CMRglu: Cerebral metabolic rate of glucose
CRLB: Cramer-Rao lower bounds
GABA: Gamma-aminobutyric acid
GC/MS: Gas chromatography/mass spectrometry
Glx: Sum of glutamate and glutamine
HES: Hydroxyethyl starch
LC/MS: Liquid chromatography/mass spectrometry
MAC: Minimal alveolar concentration
MIR: Minimum infusion rate
MRI: Magnetic resonance imaging
MRS: Magnetic resonance spectroscopy
NAA: N-acetylaspartate
NADH/NAD: Nicotinamide adenine dinucleotide
PRESS: Point resolved spectroscopy sequence
PRIS: Propofol infusion syndrome
SD: Standard deviation
TIVA: Total intravenous anesthesia
